# Putting Evidence into Practice: The *PLoS Medicine* Series on Global Mental Health Practice

**DOI:** 10.1371/journal.pmed.1001226

**Published:** 2012-05-29

**Authors:** Vikram Patel, Rachel Jenkins, Crick Lund, the PLoS Medicine Editors

**Affiliations:** 1 Faculty of Epidemiology and Population Health, London School of Hygiene & Tropical Medicine, London, United Kingdom; 2 Sangath, Goa, India; 3 Departments of Epidemiology and International Mental Health Policy, WHO Collaborating Centre, Kings College London, Institute of Psychiatry, London, United Kingdom; 4 Centre for Public Mental Health, Department of Psychiatry and Mental Health, University of Cape Town, Cape Town, South Africa; PLoS Medicine, Canada

## Abstract

The *PLoS Medicine* editors announce the launch of a new series on Global Mental Health Practice, and issue a call for papers.

Today we are delighted to announce the launch of the *PLoS Medicine* series on Global Mental Health Practice, and to issue a call for case studies that can help broaden our understanding of global mental health in “real-life” contexts.

The series was initiated by the lead author (VP), who is joined by two other leaders in global mental health (RP and CL) to serve as guest editors. Together, they bring an international, broad, and multidisciplinary perspective that will assist the *PLoS Medicine* senior Magazine editor (JC) in developing this vital series.

We aim to address the gap between public health approaches to mental health, exemplified by two series in *The Lancet* [1],[2], and clinical approaches to addressing mental disorders (such as the packages of care published in this journal [3] and efficacy studies often published in specialist psychiatric journals). Lying between these two realms is a niche for demonstrating how the principles of global mental health are put into practice in real-world contexts. These principles, reflected in the goals of international efforts such as the Movement for Global Mental Health (http://www.globalmentalhealth.org), explicitly aim to (1) improve access to evidence-based care for people with mental, neurological, or substance use disorders and (2) promote the human rights of people affected by these disorders. Articles in the *PLoS Medicine* series will report a diverse range of health interventions from around the world where action has demonstrated tangible improvements in one or both of these goals.

A key motivation for this series is to emphasize the importance of “practice-based evidence,” by placing value on the experiences and impact of interventions in real-world settings as evidence for implementation. This area is especially rich for global mental health interventions in low- and middle-income countries, which may be difficult to subject to a definitive evaluation of effectiveness such as a controlled trial. We believe that such case studies provide useful evidence, which should be disseminated widely so they can influence practice development. In particular, we are interested in interventions that are innovative and delivered in low-resource settings where the treatment gap is often largest. The series addresses the need for greater awareness of global mental health in practice, and builds on *PLoS Medicine*'s interest and leadership in global mental health [3]–[6].

## New Cases from South Africa and Afghanistan

To seed the series we have commissioned a number of case studies from around the world. Two of these are published this week in *PLoS Medicine*.

First, Simone Honikman and colleagues discuss their Perinatal Mental Health Project in Cape Town, South Africa, which developed an intervention to deliver mental health care to pregnant women in a collaborative, stepwise manner, making use of existing resources in primary care [7]. Their intervention includes training for health care workers, implementing routine antenatal screening for maternal mental distress, and establishing referral networks to on-site counselors and mental health professionals. Over three years the project achieved high levels of uptake and acceptability. Second, Peter Ventevogel and colleagues report on their efforts to integrate mental health into the health care system in Afghanistan while the system was being rebuilt from scratch [8]. Brief, practice-oriented mental health training for basic health care workers provided the opportunity to substantially increase demand for and access to mental health care services, but the authors report this opportunity also demonstrated the need for concurrent community-based approaches, capacity building, and policy development in the health care system.

## Call for Case Studies

We call for additional case studies that report global health interventions where action has demonstrated tangible improvements in one or both of the established global mental health goals: (1) to improve access to evidence-based care for people with mental, neurological, or substance use disorders and (2) to promote the human rights of people affected by these disorders. Case studies can describe interventions from any country, and should focus on (1) mental health care interventions in practice or (2) mental health policy reform or legislative change that has led to improvements in access to care and in the human rights of people with mental health conditions. Studies that describe innovative interventions delivered in low-resource settings are of particular interest. Articles that provide only descriptions of processes will not be eligible for the series, nor will case reports or case series.

The articles appear in the journal's Health in Action section (part of the *PLoS Medicine* Magazine), and authors should use our standard guidance (http://www.plosmedicine.org/static/guidelines.action#other). Articles can be up to 2,500 words long and include up to three graphics (figure, table, and/or box); these graphics do not count toward the word limits. References are limited to 30. All articles will be peer-reviewed and subject to standard *PLoS Medicine* editorial policies. Articles should follow this general format: first set the scene and provide the evidence for the intervention/project (why was it needed?); next, describe the intervention/project itself; then discuss any results of the intervention/project and the barriers and difficulties faced; finally, end by looking to the future (where is the intervention/project heading next?).

We welcome contributions from a wide variety of authors and institutions, including health activists, people affected by mental disorders, nongovernmental organizations, and researchers. We are particularly interested in featuring case studies by groups or individuals who rarely have a voice in medical journals.
